# Albumin rather than C-reactive protein may be valuable in predicting and monitoring the severity and course of acute respiratory distress syndrome in critically ill patients with or at risk for the syndrome after new onset fever

**DOI:** 10.1186/s12890-015-0015-1

**Published:** 2015-03-14

**Authors:** Sandra H Hoeboer, Heleen M Oudemans-van Straaten, AB Johan Groeneveld

**Affiliations:** Department of Intensive Care, Erasmus Medical Center, Rotterdam, The Netherlands; Departments of Intensive Care, VU University Medical Center, Amsterdam, The Netherlands

**Keywords:** Acute respiratory distress syndrome, Biomarkers, Albumin, C-reactive protein, Lactate dehydrogenase

## Abstract

**Background:**

We studied the value of routine biochemical variables albumin, C-reactive protein (CRP) and lactate dehydrogenase (LDH) to improve prediction and monitoring of acute respiratory distress syndrome (ARDS) severity in the intensive care unit.

**Methods:**

In 101 critically ill patients, with or at risk for ARDS after new onset fever, data were collected on days (D) 0, 1, 2, and 7 after inclusion. ARDS was defined by the Berlin definition and lung injury score (LIS).

**Results:**

At baseline, 48 patients had mild to severe ARDS according to Berlin and 87 according to LIS (Rs = 0.54, P < 0.001). Low baseline albumin levels were moderately associated with maximum Berlin and LIS categories within 7 days; an elevated CRP level was moderately associated with maximum Berlin categories only. The day-by-day Berlin and LIS categories were inversely associated with albumin levels (P = 0.01, P < 0.001) and directly with CRP levels (P = 0.02, P = 0.04, respectively). Low albumin levels had monitoring value for ARDS severity on all study days (area under the receiver operating characteristic curve, AUROC, 0.62-0.82, P < 0.001-0.03), whereas supranormal CRP levels performed less . When the Berlin or LIS category increased, albumin levels decreased ≥1 g/L (AUROC 0.72-0.77, P = 0.001) and CRP increased ≥104 mg/L (only significant for Berlin, AUROC 0.69, P = 0.04). When the LIS decreased, albumin levels increased ≥1 g/L (AUROC 0.68, P = 0.02). LDH was higher in 28-day non-survivors than survivors (P = 0.007).

**Conclusions:**

Overall, albumin may be of greater value than CRP in predicting and monitoring the severity and course of ARDS in critically patients with or at risk for the syndrome after new onset fever. Albumin levels below 20 g/L as well as a decline over a week are associated with ARDS of increasing severity, irrespective of its definition. LDH levels predicted 28-day mortality.

## Background

The acute respiratory distress syndrome (ARDS) is caused by alveolocapillary inflammation and increased permeability following a direct pulmonary or extrapulmonary insult. Many conditions, such as sepsis and trauma, which increase the risk for developing or worsening of ARDS are associated with fever. Fever, in turn, may aggravate alveolocapillary inflammation [1,2]. The recent Berlin definition and the old, more elaborate lung injury score (LIS) [2-8] are used to diagnose and classify ARDS. One of the drawbacks of the Berlin definition, even though moderately relating to lung edema [9], is its dependency on ventilator settings in mechanically ventilated patients (with positive end-expiratory pressure, PEEP, affecting the oxygenation ratio) and lack of a specific index of severity as the total respiratory compliance [5,7]. PEEP and compliance are incorporated in the LIS [3], which may therefore constitute a refined but more complex measure of clinical severity that correlates with alveolocapillary permeability and can be assessed at the bedside if the measurement technique is available [10,11]. The Berlin definition further includes preconditions and bilateral consolidations, even in the lowest class, while the lowest class of LIS may contain unilateral consolidation. Another limitation of these clinical classifications systems is their use of chest radiographs in the diagnostic work up. Interobserver agreement on chest imaging is poor, leading to frequent false positives and false negatives [12,13]. The systems have been compared and only partial overlap has been acknowledged [1,4]. Notably, agreement between clinical ARDS definitions and autopsy findings of diffuse alveolar damage is moderate [4,8]. Moreover, clinicians may underdiagnose ARDS, particularly when occurring late in the intensive care unit (ICU), and may be poorly able to quantify its severity and course, particularly when clinical classification systems are not commonly used [4,6-8,14].

Therefore, the search for accurate biomarkers reflecting the severity and course of alveolocapillary inflammation and increased permeability underlying the non-cardiogenic pulmonary edema of ARDS is ongoing [6,15]. We and others described that circulating albumin levels, in cross-sectional studies, inversely relates to increased alveolocapillary permeability and that hypoalbuminemia predict ARDS and edema formation in at risk patients [9,11,16-19]. Extravasation of albumin following increased permeability lowers albumin levels and the resultant low plasma colloid osmotic pressure promotes edema formation. Inflammation and injury markers such as C-reactive protein (CRP) [18,20-25] and lactate dehydrogenase (LDH) [24,26] have been suggested to help predict early onset ARDS and its outcome in cross-sectional studies. Since both clinical classifications systems allow coincident ARDS and hydrostatic edema, inflammatory markers such as CRP may be of value in separating non-hydrostatic from hydrostatic edema [15,23]. Meduri et al. [20] showed a decline in CRP and LIS in early ARDS patients responding to corticosteroids. However, the ARDS monitoring value of these routine biochemical markers, often available on a daily basis in the intensive care unit (ICU), is unknown. Associations with the severity and course of ARDS, if any, could be of value in monitoring and therefore in the management of the syndrome at the bedside.

The aim of the present study is to determine whether albumin, CRP and LDH levels are associated with the severity and course of ARDS in critically ill patients after new onset fever with or considered at risk for the syndrome defined by the Berlin and the LIS criteria. The hypothesis was that decreasing albumin and increasing CRP and LDH reflect, accurately enough for clinical use, increasing severity of ARDS if judged by both clinical classification systems. Indeed, we reasoned that the overlap of systems would be a better reference standard for potential biomarkers than either system alone.

## Methods

This was a prospective observational cohort study on the predictive and monitoring value of routine biochemical parameters for ARDS severity. The study was subsidiary to the original study on biomarkers of infection and subsequent organ failure in 101 consecutive critically ill patients with ICU-acquired fever [27]. Fever is a warning sign of inflammation. Many conditions associated with the development of ARDS, i,e, sepsis, trauma, burn injury, transfusion related lung injury amongst others, are accompanied by fever due to inflammation. The study was approved by the local Ethical Committee of the VU University Medical Center, Amsterdam. All patients or closest relatives gave written informed consent and a full description of the protocol can be found in a previous publication on this cohort evaluating biomarkers of infection only [27]. To briefly summarize: the main inclusion criterion was new onset fever: a body temperature >38.3°C measured rectally, while body temperature in the first 24 hrs of ICU stay was <37.5°C. Exclusion criteria were: age under 18 years, pregnancy, and life expectancy of <24 hours. Patients were taken care of by intensivists unaware of test results according to international and local standards. Albumin infusion was no part of standard treatment.

### Protocol

The day of new onset fever was marked day 0 (D0). Within 12 hours of meeting inclusion criteria we recorded: demographic variables, risk factors, and baseline characteristics. Disease severity was expressed by the simplified acute physiology score (SAPS) II on admission. The sequential organ failure assessment (SOFA) scores were used to monitor organ failure. Mechanical ventilation was pressure guided (control or support) and protective according to standard of care in our hospital. Chest radiographs collected on study days were reviewed by two authors (SHH and ABJG) blinded to the study results in an effort to exclude severe fluid overload or signs of congestive heart failure in classifying alveolar consolidations, In addition to the chest radiographs we used the central venous pressure (CVP), which was routinely measured in 83% of patients, to rule out severe fluid overload. The routine biochemical variables, albumin, CRP, LDH, and respiratory parameters like ventilator settings and daily chest radiographs were collected on D0, 1, 2, and 7. Total respiratory dynamic compliance was calculated from tidal volume/(plateau pressure-positive end-expiratory pressure), mL/cmH_2_O. The need for additional imaging and collection of specimen for cultures was decided upon by treating physicians blinded to study results. In case culture and/or imaging results were positive we considered the day of their collection the day of diagnosis. Sepsis is the simultaneous presence of either clinically suspected or proven infection and the systemic inflammatory response syndrome. Patients were considered suffering shock when a systolic arterial pressure <90 mmHg or a mean arterial pressure (MAP) <65 mmHg was observed for at least one hour despite adequate fluid resuscitation and/or need of vasopressor administration. All definitions, including infections, are in line with American Society of Chest Physicians/Society of Critical Care Medicine criteria [28,29]. For the sake of clarity, pneumonia is either community-, hospital- or ventilator-acquired. To define ARDS severity on study days, both the Berlin definition and the LIS were used. The Berlin definition divides patients into 4 categories that reflect the severity of the syndrome: no ARDS (Berlin 0, not fulfilling preconditions or P_a_O_2_/F_I_O_2_ > 300 mmHg), mild ARDS (Berlin 1, 200 mmHg < P_a_O_2_/F_I_O_2_ ≤ 300 mmHg), moderate ARDS (Berlin 2, 100 mm Hg < P_a_O_2_/F_I_O_2_ ≤ 200 mm Hg), and severe ARDS (Berlin 3, P_a_O_2_/F_I_O_2_ ≤ 100 mmHg). Patients suffer from ARDS if its onset is within 1 week of a known clinical insult or worsening of respiratory symptoms, there are bilateral opacities on chest radiograph not fully explained by cardiac failure of fluid overload, and the PEEP level is ≥5 cmH_2_O [7]. We also calculated the LIS [3]; an average based on classification of patients by the number of quadrants with alveolar consolidation on the anterior-posterior chest radiograph, severity of hypoxemia, pulmonary compliance (tidal volume/(peak inspiratory pressure-PEEP)), and PEEP level. We used the lowest P_a_O_2_/F_I_O_2_ measured on study days and recorded the corresponding PEEP levels and compliance at the time of sampling. Based on their LIS, patients were divided into three categories that reflect disease severity: no lung injury (LIS ≤1), mild ARDS (LIS 1-2.5), and severe ARDS (LIS >2.5) [6]. Follow up was until day 28 and we checked the clinical state or date of death for all patients.

### Biochemistry

Albumin was measured by using Albumin/BCP (Roche Diagnostics, Mannheim, Germany); normal values are 35-47 g/L. CRP was measured using an immunoturbidimetric assay by Modular analytics < P > Roche diagnostics (Mannheim, Germany) and normal values are <5 mg/L. LDH was measured using lactate dehydrogenase optimized (Roche diagnostics, Mannheim, Germany); the normal range is 240- 480 U/L.

### Statistical analysis

Data are expressed as median (inter quartile range) or number (percentage) where appropriate. Non-normally distributed data were logarithmically transformed where appropriate. To study group differences in continuous variables we performed the Kruskal-Wallis test followed by a Mann-Whitney U test and for categorical variables we used the X^2^ test. We used the Spearman’s rank correlation for non-normally distributed data to indicate any overlap between the Berlin and LIS categories. First, to evaluate the diagnostic value of day 0 routine biochemical variable levels for the maximum ARDS severity within one week after inclusion, we calculated the area under the receiver operating characteristic curve (AUROC) and associated statistical predictive variables, such as optimal cutoff values, sensitivity, specificity, positive and negative predictive values. We performed the AUROC analyses using MedCalc for Windows, version 13 (MedCalc Software, Ostend, Belgium). The optimal diagnostic cutoff value was derived from the optimal Youden’s index ( J = sensitivity + specificity-1; were J = 1 represents perfect diagnostic test accuracy, ref [30]). Prior to data-analysis and in line with the literature we decided that an AUROC >0.65 was clinically relevant and >0.70 of good discriminative value. Subsequently, to study the monitoring value of routine biochemical markers for ARDS longitudinally, we performed generalized estimating equations (GEE), taking repeated measures in the same patient and first order interactions into account. To further study the monitoring value of the biochemical markers for ARDS severity, we calculated the AUROCs on individual study days. Finally, we compared the change in biomarker levels (increase or decrease) over 7 days between patients with increasing, equal or decreasing ARDS severity. To study this association we calculated the day 0 to day 7 change in routine biochemical variables (∆ = D0-7) and the change in Berlin and LIS category and tested for differences between groups. We compared routine biochemical variable levels between 28-day survivors and non-survivors and between 28-day survivors and non-survivors with a maximum Berlin ≥1 or maximum LIS >1. Since LDH did not appear useful in diagnosing ARDS severity and course, associations with outcome are reported only. All tests were two-sided and P-values ≤0.05 were considered statistically significant. Exact P values are given, unless <0.001.

## Results

### Patients

**Figure 1:**
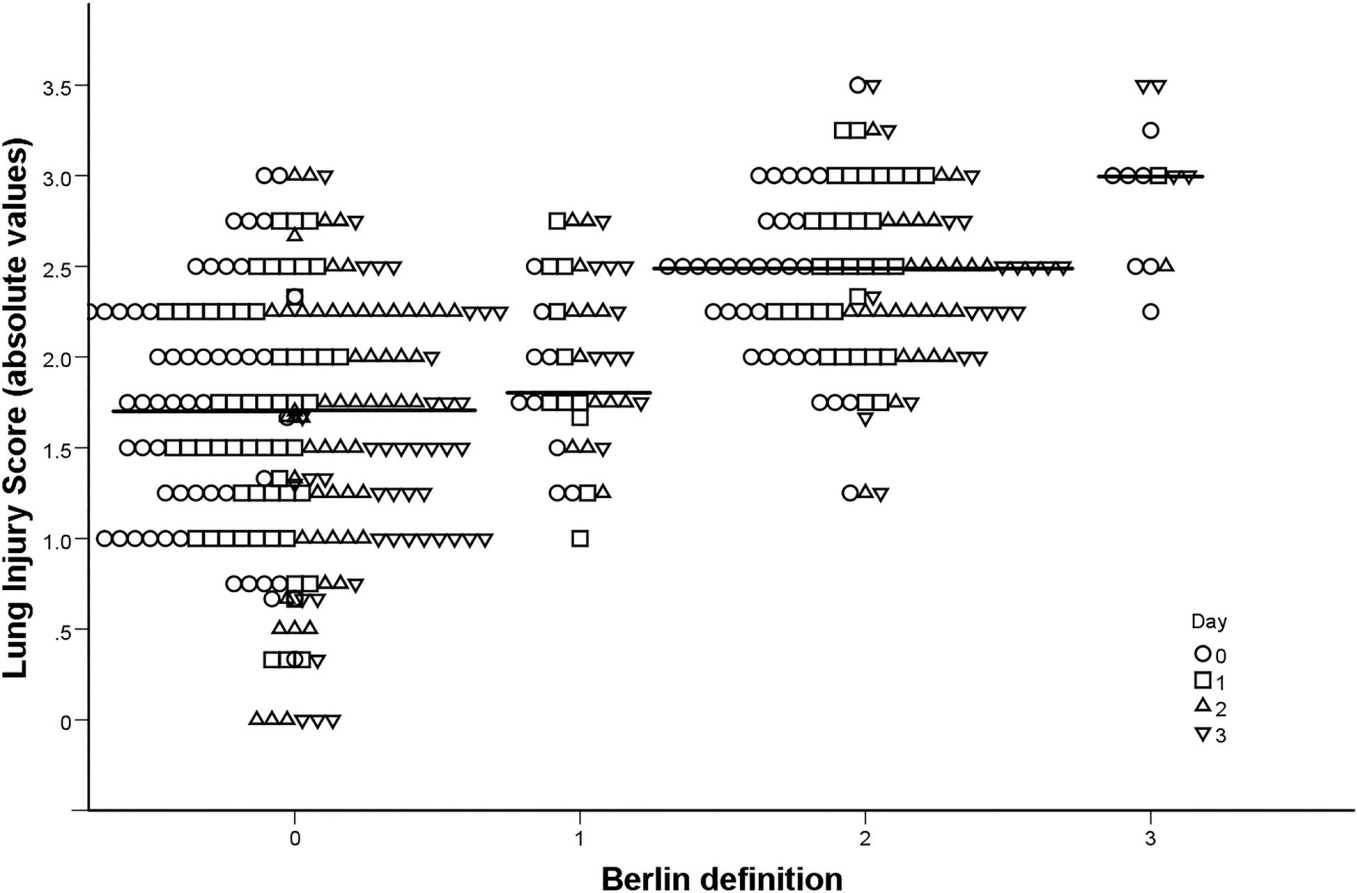
**Scatterplot of the Berlin definition categories vs. the lung injury score of ARDS (R**_**s**_ **= 0.54, P < 0.001).**

Baseline patient characteristics according to Berlin categories are presented in Table 1. Of the 101 patients, 53 (52%) had no ARDS on D0, 9 (9%) mild ARDS, 32 (32%) moderate, and 7 (7%) severe ARDS. In patients with severe ARDS (Berlin 3), SOFA scores were higher than in those without ARDS (Berlin 0, P = 0.02). The P_a_O_2_/F_I_O_2_ ratio in patients without ARDS (Berlin 0) was lower than in those with mild ARDS (Berlin 1, P = 0.001), but higher than in Berlin categories 2 (P = 0.05) and 3 (P < 0.001) (Table 2). Despite the relatively low P_a_O_2_/F_I_O_2_ ratio in the Berlin 0 category these patients did not fulfill the other prerequisites for ARDS. Similar variables are presented for the LIS categories on D0 in Table 1. According to the LIS, 14 (14%) patients had no ARDS on D0, 69 (68%) mild, and 18 (18%) severe ARDS. In comparison to patients without lung injury, patients with mild (LIS >1.0) or severe ARDS (LIS >2.5) were more likely to need mechanical ventilation (P = 0.001 and P = 0.02), required more ventilator days (P = 0.04 and P = 0.03), and had a higher D0 SOFA score (P = 0.02 and P = 0.001; Table 2). On the day of inclusion an ARDS risk factor (Table 3) was present in 93% of Berlin ARDS patients and 96% of LIS ARDS patients, while some patients suffered from more than one risk factor. The correlation between the Berlin and LIS categories was moderate (Rs = 0.54, P < 0.001) (Figure 1). Forty-one patients had a Berlin category <1 and 6 patients had a LIS ≤1 throughout the study.

**Table 1:** **Patient characteristics according to Berlin and LIS categories of ARDS at baseline**

**Berlin category**	**0**	**1**	**2**	**3**	**P-value**
	**N = 53**	**N = 9**	**N = 32**	**N = 7**	
Age, years	61 (30)	71 (22)	63 (24)	69 (29)	0.21
Sex, male	39 (74)	5 (56)	20 (63)	5 (71)	0.60
SAPS II admission	46 (20)	59 (24)	49 (16)	44 (57)	0.39
SOFA D0	7 (4)	8 (5)	9 (5)	10 (5)	0.06
ICU days until inclusion	6 (12)	7 (19)	8 (12)	9 (32)	0.98
CVP D0, mmHg	9 (5)	5 (2)	6 (6)	7 (3)	0.26
CVP D1, mmHg	8 (5)	6 (5)	7 (4)	6 (0)	0.83
CVP D2, mmHg	7 (4)	9 (2)	6 (4)	5 (0)	0.25
CVP D7, mmHg	7 (4)	9 (4)	7 (5)	9 (1)	0.49
Vasopressor use D0-7	28 (53)	6 (67)	23 (72)	5 (71)	0.20
Renal replacement therapy D0-7	3 (6)	1 (11)	4 (13)	0	0.57
Albumin 20% administration (100 mL) D0-7	3 (6)	2 (22)	8 (25)	0	0.03
Corticosteroids use D -7-0	23 (43)	5 (56)	14 (44)	3 (43)	0.92
Corticosteroid use D 0-7	23 (43)	7 (78)	16 (50)	4 (57)	0.28
28-day mortality	9 (17)	4 (44)	10 (31)	3 (43)	0.15
**LIS category**	**LIS <1**	**LIS 1.0-2.5**	**LIS >2.5**		
	**N =14**	**N = 69**	**N =18**	**P-value**	
Age, years	62 (28)	63 (24)	59 (28)	0.92	
Sex, man	10 (71)	47 (68)	12 (67)	0.96	
SAPS II at admission	49 (20)	47 (20)	45 (23)	0.35	
SOFA D0	5 (2)	8 (5)	10 (3)	0.004	
ICU days until inclusion	6 (14)	7 (9)	6 (12)	0.85	
CVP D0, mmHg	8 (6)	7 (5)	8 (5)	0.79	
CVP D1, mmHg	7 (4)	7 (6)	7 (4)	0.50	
CVP D2, mmHg	3 (0)	7 (3)	7 (6)	0.32	
CVP D7, mmHg	6 (7)	7 (5)	8 (3)	0.61	
Vasopressor use D0-7	5 (39)	4 (63)	14 (82)	0.05	
Renal replacement therapy D0-7	0	7 (10)	1 (6)	0.40	
Albumin 20% administration (100 mL) D 0-7	0	12 (17)	1 (6)	0.12	
Corticosteroids use D -7-0	6 (43)	31 (45)	8 (44)	0.99	
Corticosteroid use D 0-7	4 (29)	35 (51)	11 (61)	0.18	
28-day mortality	2 (14)	18 (26)	6 (33)	0.47	

Median (interquartile range) or number (percentage), where appropriate. Abbreviations: ARDS- acute respiratory distress syndrome; CPR- cardiopulmonary resuscitation; CVP-central venous pressure; D-day; ICU-intensive care unit; P_a_O_2_/F_I_O_2_-arterial O_2_ pressure over inspiratory O_2_ fraction; PEEP-positive end-expiratory pressure; SAPS-simplified acute physiology score; SOFA-sequential organ failure assessment.

**Table 2:** **Ventilator course between days 0 and 7 according to Berlin and LIS categories of ARDS**

**Berlin category**	**0**	**1**	**2**	**3**	**P-value**
	**N = 53**	**N = 9**	**N = 32**	**N = 7**	
**Ventilator course D0-7**					
Mechanical ventilation D0	47 (89)	9 (100)	32 (100)	7 (100)	0.12
duration, days	22 (30)	23 (27)	22 (25)	16 (26)	0.85
P_a_O_2_/F_I_O_2_ ratio D0	180 (76)	226 (60)	155 (49)	89 (32)	<0.001
P_a_O_2_/F_I_O_2_ ratio D1	191 (66)	208 (64)	156 (34)	91 (0)	<0.001
P_a_O_2_/F_I_O_2_ ratio D2	194 (105)	252 (41)	168 (35)	68 (0)	<0.001
P_a_O2/F_I_O_2_ ratio D7	189 (110)	239 (23)	161 (48)	73 (21)	<0.001
PEEP D0, cmH_2_0	8 (7)	8 (4)	10 (4)	10 (2)	0.10
PEEP D1, cmH_2_0	8 (7)	8 (6)	10 (4)	13 (0)	0.13
PEEP D2, cmH_2_0	8 (7)	10 (6)	10 (4)	8 (0)	0.40
PEEP D7, cmH_2_0	6 (6)	11 (7)	9 (4)	13 (8)	0.001
Compliance D0, mL/cmH_2_0	32 (23)	39 (13)	38 (23)	36 (27)	0.88
Compliance D1, mL/cmH_2_0	39 (18)	35 (24)	35 (18)	21 (0)	0.24
Compliance D2, mL/cmH_2_0	35 (20)	40 (30)	35 (20)	33 (0)	0.78
Compliance D7, mL/cmH_2_0	47 (34)	31 (12)	37 (24)	19 (9)	0.11
Tidal volume D0, mL	500 (217)	500 (116)	520 (206)	530 (256)	0.92
Tidal volume D1, mL	520 (120)	500 (140)	500 (210)	530 (150)	0.68
Tidal volume D2, mL	520 (123)	500 (147)	505 (250)	550 (300)	0.78
Tidal volume D7, mL	530 (192)	463 (216)	500 (150)	450 (250)	0.17
Chest radiograph D0, quadrants	1 (1)	2 (0)	2 (1)	2 (1)	<0.001
Chest radiograph D1, quadrants	1 (1)	2 (0)	2 (1)	2	<0.001
Chest radiograph D2, quadrants	1 (1)	2 (1)	2 (1)	3	<0.001
Chest radiograph D7, quadrants	0 (1)	2 (0)	2 (2)	3 (1)	<0.001
**LIS category**	**LIS <1**	**LIS 1.0-2.5**	**LIS >2.5**		
	**N =14**	**N = 69**	**N =18**	**P-value**	
**Ventilator course D0-7**					
Mechanical ventilation D0	10 (71)	67 (97)	18 (100)	0.001	
duration, days	11 (20)	22 (27)	28 (21)	0.07	
P_a_O_2_/F_I_O_2_ ratio D0	214 (130)	174 (68)	112 (70)	<0.001	
P_a_O_2_/F_I_O_2_ ratio D1	238 (157)	184 (60)	149 (40)	<0.001	
P_a_O_2_/F_I_O_2_ ratio D2	284 (111)	181 (72)	156 (48)	<0.001	
P_a_O_2_/F_I_O_2_ ratio D7	269 (162)	177 (79)	102 (78)	<0.001	
PEEP D0, cmH_2_0	5 (2)	9 (6)	14 (3)	<0.001	
PEEP D1, cmH_2_0	5 (1)	9 (4)	13 (4)	<0.001	
PEEP D2, cmH_2_0	5 (1)	9 (5)	12 (6)	<0.001	
PEEP D7, cmH_2_0	4 (3)	8 (6)	14 (5)	<0.001	
Compliance D0, mL/cmH_2_0	44 (57)	39 (20)	27 (18)	<0.001	
Compliance D1, mL/cmH_2_0	51 (25)	37 (18)	28 (21)	<0.001	
Compliance D2, mL/cmH_2_0	61 (105)	35 (19)	31 (21)	0.01	
Compliance D7, mL/cmH_2_0	65 (75)	37 (24)	20 (11)	0.007	
Tidal volume D0, mL	409 (268)	500 (176)	550 (154)	0.39	
Tidal volume D1, mL	523 (177)	500 (166)	523 (95)	0.92	
Tidal volume D2, mL	490 (138)	525 (133)	535 (194)	0.43	
Tidal volume D7, mL	450 (150)	500 (213)	500 (138)	0.71	
Chest radiograph D0, no quadrants	0 (1)	2 (1)	2 (3)	<0.001	
Chest radiograph D1, no quadrants	1 (1)	1 (1)	2 (2)	<0.001	
Chest radiograph D2, no quadrants	0 (1)	2 (1)	2 (3)	<0.001	
Chest radiograph D7, no quadrants	1 (0)	2 (1)	2 (1)	0.005	

Median (interquartile range) or number (percentage), where appropriate. Abbreviations: ARDS- acute respiratory distress syndrome; D-day; ICU-intensive care unit; P_a_O_2_/F_I_O_2_-arterial O_2_ pressure over inspiratory O_2_ fraction; PEEP-positive end-expiratory pressure.

**Table 3:** **ARDS risk factors on ICU admission and on study inclusion**

**Berlin category**	**0**	**1**	**2**	**3**	**P-value**
	**N = 53**	**N = 9**	**N = 32**	**N = 7**	
**ARDS risk factors on ICU admission**					
Sepsis	14 (26)	4 (44)	12 (38)	1 (14)	0.42
Shock	8 (15)	3 (33)	9 (28)	0	0.18
Trauma	11 (21)	0	2 (6)	0	0.09
General surgery	30 (57)	3 (33)	16 (50)	5 (71)	0.43
Vascular surgery	4 (8)	1 (1)	3 (9)	2 (29)	0.38
Cardiac surgery	3 (6)	0	2 (6)	1 (14)	0.69
Intracranial bleeding	10 (19)	2 (22)	1 (3)	1 (14)	0.19
CPR	6 (11)	1 (11)	2 (6)	1 (14)	0.86
Other	7 (13)	0	2 (6)	0	0.38
**ARDS risk factors on D0**					
Sepsis	29 (56)	4 (44)	13 (41)	4 (57)	0.66
Shock	14 (26)	5 (56)	14 (44)	4 (57)	0.13
Pneumonia	3 (6)	0	4 (13)	2 (29)	0.14
Aspiration pneumonia	3 (6)	0	0	0	0.43
Peritonitis	3 (6)	1 (11)	1 (3)	0	0.71
Infected pancreatitis	2 (4)	0	1 (3)	0	0.89
Miscellaneous infection	20 (38)	2 (22)	10 (31)	3 (43)	0.75
Surgery within 48 hrs prior to inclusion	8 (15)	1 (11)	4 (13)	0	0.73
**LIS category**	**LIS ≤1.0**	**LIS 1.0-2.5**	**LIS >2.5**	**P-value**	
	**N =14**	**N = 69**	**N =18**		
**ARDS risk factors on ICU admission**					
Sepsis	3 (21)	22 (32)	6 (33)	0.72	
Shock	2 (14)	17 (25)	1 (6)	0.17	
Trauma	1 (7)	10 (15)	2 (11)	0.73	
General surgery	8 (57)	37 (54)	9 (50)	0.92	
Vascular surgery	0	8 (11)	2 (11)	0.41	
Cardiac surgery	0	4 (6)	2 (11)	0.42	
Intracranial bleeding	5 (36)	7 (10)	2 (11)	0.04	
CPR	1 (7)	7 (10)	2 (11)	0.93	
Other	3 (21)	5 (7)	1 (6)	0.20	
**ARDS risk factors on D0**					
Sepsis	9 (64)	34 (49)	7 (39)	0.36	
Shock	2 (14)	27 (39)	8 (44)	0.16	
Pneumonia	1 (7)	6 (9)	2 (11)	0.92	
Aspiration pneumonia	1 (7)	1 (2)	1 (6)	0.41	
Peritonitis	1 (7)	3 (4)	1 (6)	0.90	
Infected pancreatitis	2 (14)	1 (1)	0	0.03	
Miscellaneous infection	7 (50)	25 (36)	3 (17)	0.13	
Surgery within 48 hrs prior to inclusion	1 (7)	9 (13)	3 (17)	0.73	

Number (percentage). Abbreviations: ARDS-acute respiratory distress syndrome; CPR-cardiopulmonary resuscitation; ICU-intensive care unit; hrs- hours; LIS-lung injury.

### Association with ARDS severity

Table 4 shows some associative values of D0 albumin and CRP for the maximum Berlin and LIS categories within one week after inclusion. During the week, 42 patients reached a maximum Berlin <1 and 59 patients a maximum Berlin ≥1, whereas 6 patients reached a maximum LIS ≤1 and 95 patients a maximum LIS >1. Patients with a maximum Berlin ≥1 reached their maximum Berlin score after day 0 in 30% of cases. Patients with a maximum LIS >1 reached their maximum LIS score after day 0 in 26% of cases. The associative values of albumin ranged between (AUROC) 0.62 to 0.65 (P = 0.04 or lower). An albumin level <20 g/L was associated with a maximum Berlin category ≥1 and albumin <22 g/L was associated with a maximum LIS >2.5. In contrast, CRP levels >138 mg/L were associated with a maximum Berlin category ≥2 while CRP levels >81 mg/L were associated with a maximum LIS >1.

**Table 4:** **Diagnostic values of D0 albumin and CRP for maximum Berlin and LIS categories within one week after new onset fever in critically ill patients**

	**AUROC**	**95% CI**	**P-value**	**Optimal cutoff**	**SN**	**SP**	**PPV**	**NPV**
**Maximum Berlin ≥1 (N = 59)**								
Albumin	0.65	0.53-0.76	0.01	<20 g/L	71	58	71	58
**Maximum Berlin ≥2 (N = 50)**								
Albumin	0.63	0.52-0.74	0.02	<20 g/L	72	53	61	65
CRP	0.62	0.51-0.74	0.03	>138 mg/L	54	76	69	62
**Maximum LIS >1.0 (N = 95)**								
CRP	0.82	0.64-1.00	0.002	>81 mg/L	77	80	99	15
**Maximum LIS >2.5 (N = 34)**								
Albumin	0.62	0.51-0.73	0.04	<22 g/L	91	31	41	87

Abbreviations: AUROC-area under the curve; ARDS-acute respiratory distress syndrome; CI-confidence interval; CRP-C-reactive protein; LIS- lung injury score; NPV-negative predictive value; PPV-positive predictive value; SN-sensitivity; SP- specificity.

### Monitoring ARDS severity

**Figure 2:**
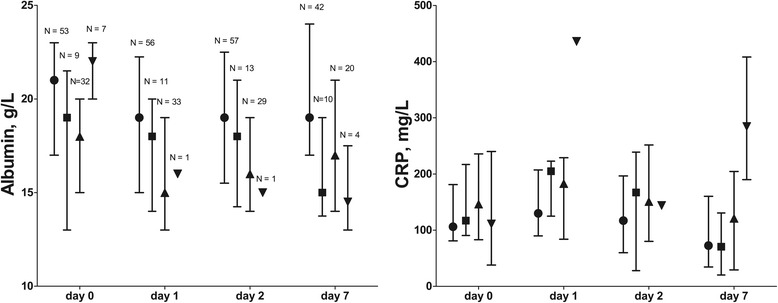
**Median and inter quartile range of albumin and C-reactive protein (CRP) for the Berlin definition on ARDS.** The Berlin categories are inversely associated with albumin levels (P = 0.01) and directly with CRP levels (P = 0.02) in generalized estimating equations. ● no acute respiratory distress syndrome (ARDS, Berlin 0), ■ mild ARDS (Berlin 1), ▲ moderate ARDS (Berlin 2) ▼ severe ARDS (Berlin 3). Numbers refer to numbers of patients.

**Figure 3:**
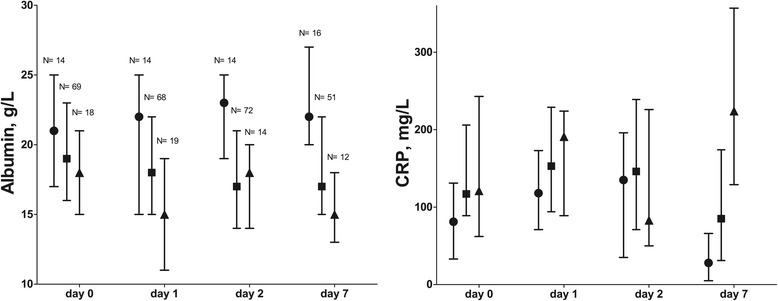
**Median and inter quartile range of albumin and C-reactive protein (CRP) for the lung injury score (LIS).** The LIS categories are inversely associated with albumin (P < 0.001) and directly with CRP (P = 0.04) in generalized estimating equations. ● no lung injury (LIS ≤1.0), ■ mild acute respiratory distress syndrome ARDS (LIS 1.0-2.5), ▲ severe ARDS (LIS >2.5). Numbers refer to numbers of patients.

**Figure 4:**
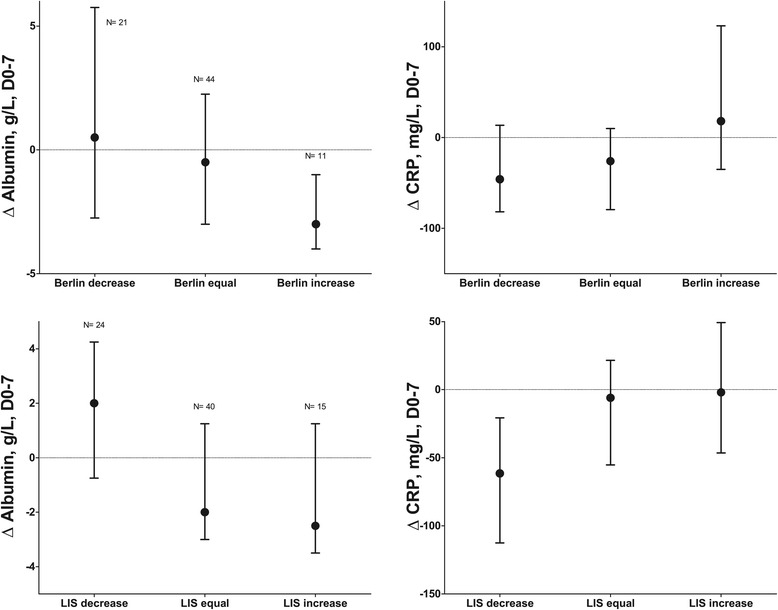
**Changes of albumin and C-reactive protein (CRP) levels for changes in Berlin and lung injury score (LIS) categories between D0-7.** The change in albumin levels is associated with a change in Berlin and LIS category (P = 0.05 and P = 0.03, respectively). A change in CRP levels is associated with a change in LIS category only (P = 0.03).

Figure 2 presents values according to Berlin categories and Figure 3 according to LIS categories in the course of time. Of note, changing numbers per day indicate that ARDS was deteriorating or improving over time in some patients. Albumin levels were lower and CRP levels were higher with increasing Berlin and LIS category. The albumin levels had a monitoring value, albeit moderate, on all study days and cutoff values generally decreased with increasing ARDS severity (AUROC between 0.62-0.82, P < 0.001-0.03, Table 5). CRP levels had less frequent monitoring value for ARDS severity. Figure 4 depicts the change in albumin and CRP levels between D0 and 7 (∆D0-7) in relation to the change in Berlin and LIS category: albumin levels inversely related to change in ARDS severity regardless of definition. Increasing CRP levels were associated with increasing Berlin definition only. A decrease in albumin of ≥1 g/L and an increase of CRP ≥104 mg/L were associated with an increase in ARDS severity by Berlin category (AUROC 0.72, P = 0.001 with sensitivity 100, specificity (SP) 42, positive predictive value (PPV) 23 and negative predictive value (NPV) 100%; AUROC 0.69, P = 0.04, SN 27, SP 98, PPV 78 and NPV 88%, respectively). A decrease in albumin ≥1 g/L was associated with an increase in LIS category (AUROC 0.77, P < 0.001, SN 91, SP 54, PPV 26, NPV 97), and an increase in albumin ≥1 g/L with a decrease in LIS category (AUROC 0.68, P = 0.02, SN 61, SP 73, PPV 42, NPV 85).

**Table 5:** **Monitoring values for ARDS severity on study days**

		**AUROC**	**95% CI**	**P-value**	**Optimal cutoff**	**SN**	**SP**	**PPV**	**NPV**
**Berlin ≥1**	**day 0**								
	Albumin	0.62	0.52-0.72	0.03	<20 g/L	71	51	58	65
	**day 1**								
	Albumin	0.66	0.56-0.77	0.003	<20 g/L	84	44	55	77
	**day 2**								
	Albumin	0.67	0.56-0.78	0.002	<17 g/L	67	65	58	73
	**day 7**								
	Albumin	0.71	0.59-0.81	<0.001	<14 g/L	38	92	81	63
**Berlin ≥2**	**day 1**								
	Albumin	0.67	0.57-0.76	0.002	<18 g/L	73	54	44	80
	**day 2**								
	Albumin	0.68	0.58-0.77	<0.001	<17 g/L	73	62	46	84
	**day 7**								
	CRP	0.65	0.53-0.76	0.045	>105 mg/L	67	64	47	80
**Berlin ≥3**	**day 7**								
	Albumin	0.77	0.65-0.86	0.01	<18 g/L	100	49	10	100
	CRP	0.91	0.82-0.97	<0.001	>162 mg/L	100	73	19	100
**LIS >1**	**day 0**								
	CRP	0.70	0.60-0.79	0.01	>81 mg/L	78	54	92	27
	**day 1**								
	CRP	0.65	0.55-0.75	0.04	>182 mg/L	42	92	97	20
	**day 2**								
	Albumin	0.82	0.73-0.89	<0.001	<21 g/L	86	64	94	43
	**day 7**								
	Albumin	0.81	0.70-0.89	<0.001	<17 g/L	56	92	97	31
	CRP	0.79	0.68-0.87	<0.001	>60 mg/L	68	79	93	36
**LIS >2.5**	**day 1**								
	Albumin	0.69	0.56-0.78	0.02	<11 g/L	39	96	70	88
	**day 7**								
	Albumin	0.72	0.60-0.82	0.004	<18 g/L	83	53	26	94
	CRP	0.83	0.73-0.91	<0.001	>158 mg/L	75	80	41	94

Abbreviations: AUROC-area under the receiver operating characteristics curve; CI-confidence interval; CRP-C-reactive protein; LIS-lung injury score; NPV-negative predictive value; PPV-positive predictive value; SN-sensitivity-SP-specificity.

### Mortality

In 28-day non-survivors, D2 and peak LDH levels were higher (647 (5005) and 756 (409) U/L, respectively, P = 0.003) than in survivors (435 (199) and 543 (362) U/L, respectively, P = 0.007). In patients with ARDS according to the Berlin definition, LDH levels were higher in non-survivors (665 (421) U/L) than in survivors (458 (243) U/L) on D1 (P = 0.03), in non-survivors (706 (621) U/L) than in survivors (452 (225) U/L) on D2 (P < 0.001), and in non-survivors (618 (364) U/L) than in survivors (454 (258) U/L) on D7 (P = 0.02). Peak LDH levels in non-survivors (876 (653) U/L) were higher than in survivors (581 (347) U/L, P = 0.002). In patients with ARDS according to the LIS, peak LDH levels in non-survivors (756 (409) U/L) were higher than in survivors (548 (359) U/L, P = 0.009). Albumin and CRP did not have prognostic significance.

## Discussion

This longitudinal study in critically ill patients with or at risk for ARDS with new onset fever suggests that albumin rather than CRP levels are valuable in daily monitoring of ARDS severity and course at the bedside. Although the associative values were only moderate, a low albumin was a useful indicator on all study days, while a supranormal CRP cutoff was less frequently associated with ARDS severity. During the week, a change in albumin levels was inversely related to a change in ARDS severity regardless of definition. In contrast, increasing CRP levels were associated with increasing Berlin categories only. The LDH levels only predicted 28-day mortality.

Only partial overlap between Berlin and LIS categories has been observed before [1,4]. In the absence of a reference standard like autopsy or measurement of alveolar-capillary permeability, we cannot determine whether the Berlin categories underestimated or the LIS overestimated the severity of ARDS. A relatively high P_a_O_2_/F_I_O_2_ ratio, in the presence of relatively high PEEP, may not meet Berlin criteria if preconditions and bilaterality are absent, whereas PEEP adds to the LIS score [3]. The sensitivity of compliance, which is often the first parameter to deteriorate after initiation of lung injury, even before onset of edema, could also explain the higher frequency of ARDS by LIS than Berlin definitions [31]. The Berlin definition includes bilateral chest radiograph abnormalities, while the LIS includes quadrants. However, chest radiographs have high interobserver variability, leading to frequent false positives and negatives [12,13]. As such the LIS may constitute a more sensitive measure of the clinical severity of ARDS correlating with alveolocapillary permeability than the Berlin definition, but thereby carries the risk of oversensitivity and overestimation [1,5], In any case, the CVP was comparable between Berlin and LIS categories, so it is less likely that severe fluid overload explains the difference in ARDS rating between definitions. Otherwise, the rate and distribution of risk factors in this population with or at risk for late ARDS in the ICU is in agreement with the literature, showing ICU-acquired sepsis as the leading cause (Table 3) [1,14]. The relatively high ARDS prevalence reflects the selection of critically ill patients with new onset fever, suggesting new onset sepsis or inflammation both important ARDS risk factors.

We reasoned that an association with both ARDS severity classifications would render a potential biomarker clinically valuable, in the absence of a true reference standard of ARDS. Albumin levels had monitoring value for ARDS defined by the Berlin definition and the LIS on all study days and cutoff values in AUROC’s declined as disease severity increased. This agrees with the idea that a low albumin is indeed involved in ARDS pathogenesis, i.e. increased permeability edema, as suggested before in cross-sectional studies [9,11,16-19]. Albumin levels did not prognosticate outcome as in other studies [18]. CRP levels had no consistent monitoring ability for ARDS. A supranormal CRP was mainly associated with severe ARDS on D7. Our data suggest that CRP is not useful as a marker of ARDS severity and course, in line with some studies [22,24]. However, in previous studies CRP had value in differentiating ARDS from cardiogenic pulmonary edema [23] and the CRP and LIS decline upon successful ARDS treatment by corticosteroids [20]. In our study, patients with cardiogenic edema were excluded. The use of corticosteroids on clinical indication could have been a confounder but distribution between ARDS categories was comparable. CRP levels did not prognosticate outcome in our study in line with some [22], but in contrast to reports on the association between elevated CRP levels and survival [21] or non-survival [25]. Even though ARDS can be considered an inflammatory response of the lung, numerous other factors can be responsible for elevated CRP levels in critically ill patients. The levels of LDH, a marker of cell damage, were not diagnostic of ARDS severity and course in line with some [24], but in contrast to other observations suggesting elevated levels in sepsis patients progressing to ARDS [26]. The LDH levels were however associated with 28-day mortality, which has not been reported before.

A limitation of this study is its relatively small sample size and heterogeneous population. Considering generalizabilty of the results the latter might be an advantage as well. We included patients with the symptom fever rather than with specific conditions to focus on an inflammatory response as a major risk factor for developing or worsening ARDS. Few patients received corticosteroids or albumin as part of their treatment, but their distribution was equal between ARDS categories and therefore do not invalidate our conclusions. With exceptions, the AUROC’s were generally not >0.75. Low predictive capacity could also be related to the inclusion of high risk patients only. This must be weighed against the accessibility of these variables which are collected almost daily and routinely in many ICU’s. Nevertheless, even though the associations between albumin levels and ARDS were modest, they were present on all individual study days and over the course of a week. Furthermore, albumin was inversely related to disease severity regardless of the clinical definition and its course predicted disease course (AUROC 0.68-0.77 respectively), while neither albumin nor CRP had any predictive value for 28-day mortality, possibly due to the limited power of this study. Our study suggests that albumin levels may have practical value in monitoring the severity of ARDS at the bedside of critically ill patients without the need for LIS calculations which are hardly done routinely. Assessing the P_a_O_2_/F_I_O_2_ ratio and chest radiograph may be insufficient to monitor ARDS, since both are treatment-dependent, for instance with higher P_a_O_2_/F_I_O_2_ ratios and more aerated chest radiographs with higher PEEP. Even though two authors reviewed clinical history, chest radiographs, and CVP to exclude severe fluid overload or congestive heart failure in classifying alveolar consolidations we cannot fully exclude a a component of hydrostatic edema in some of our ARDS patients. Nevertheless, even when there is dilution due to fluid administration hypoalbuminemia leads to lowered oncotic pressure and in the presence of increased vascular permeability this leads to pulmonary edema and ARDS. As shown by others low total protein and albumin levels, regardless of fluid state, are associated with the presence and development of ARDS [11,16,17]. Our study adds to the latter studies by focusing on the value of albumin in late ARDS (85-90% after 48 hours, depending on definition) in the ICU, a commonly underdiagnosed condition [14].

## Conclusions

Overall, albumin rather than CRP may be valuable in predicting and monitoring the severity and course of ARDS in febrile critically patients with or at risk for the syndrome. Albumin levels below 20 g/L as well as a decline in albumin levels are associated with ARDS of increasing severity, irrespective of definition. LDH levels predicted 28-day mortality but had no monitoring value for ARDS severity.

## Abbreviations

ARDS: Acute respiratory distress syndrome
AUROC: Area under the receiver operating characteristic curve
CI: Confidence interval
CPR: Cardiopulmonary resuscitation
CRP: C-reactive protein
CVP: Central venous pressure
D: Day
ICU: Intensive care unit
LDH: Lactate dehydrogenase
LIS: Lung injury score
NPV: Negative predictive value
P_a_O_2_/F_I_O_2_: Arterial O_2_ pressure over inspiratory O_2_ fraction
PEEP: Positive end-expiratory pressure
PPV: Positive predictive value
SAPS: Simplified acute physiology score
SN: Sensitivity
SOFA: Sequential organ failure assessment
SP: Specificity
